# Genetic association of zinc transporter 8 (ZnT8) autoantibodies in type 1 diabetes cases

**DOI:** 10.1007/s00125-012-2540-2

**Published:** 2012-04-12

**Authors:** J. M. M. Howson, S. Krause, H. Stevens, D. J. Smyth, J. M. Wenzlau, E. Bonifacio, J. Hutton, A. G. Ziegler, J. A. Todd, P. Achenbach

**Affiliations:** 1 Juvenile Diabetes Research Foundation/Wellcome Trust Diabetes and Inflammation Laboratory, Cambridge Institute for Medical Research, Department of Medical Genetics, University of Cambridge, Addenbrooke’s Hospital, Hills Road, Cambridge, CB2 0XY UK; 2 Forschergruppe Diabetes, Munich University of Technology, Munich, Germany; 3 Barbara Davis Diabetes Centre, University of Colorado, Denver, CO USA; 4 Centre for Regenerative Therapies, Dresden University of Technology, Dresden, Germany; 5 Institute of Diabetes Research, Helmholtz Centre Munich, Neuherberg, Germany

**Keywords:** Autoantibody, *FCRL3*, HLA, MHC, *SLC30A8*, Type 1 diabetes, Zinc transporter 8

## Abstract

**Aims/hypothesis:**

Autoantibodies to zinc transporter 8 (ZnT8A) are associated with risk of type 1 diabetes. Apart from the *SLC30A8* gene itself, little is known about the genetic basis of ZnT8A. We hypothesise that other loci in addition to *SLC30A8* are associated with ZnT8A.

**Methods:**

The levels of ZnT8A were measured in 2,239 British type 1 diabetic individuals diagnosed before age 17 years, with a median duration of diabetes of 4 years. Cases were tested at over 775,000 loci genome wide (including 53 type 1 diabetes associated regions) for association with positivity for ZnT8A. ZnT8A were also measured in an independent dataset of 855 family members with type 1 diabetes.

**Results:**

Only *FCRL3* on chromosome 1q23.1 and the HLA class I region were associated with positivity for ZnT8A. rs7522061T>C was the most associated single nucleotide polymorphism (SNP) in the *FCRL3* region (*p* = 1.13 × 10^−16^). The association was confirmed in the family dataset (*p* ≤ 9.20 × 10^−4^). rs9258750A>G was the most associated variant in the HLA region (*p* = 2.06 × 10^−9^ and *p* = 0.0014 in family cases). The presence of ZnT8A was not associated with *HLA-DRB1*, *HLA-DQB1*, *HLA-A*, *HLA-B* or *HLA-C* (*p* > 0.05). Unexpectedly, the two loci associated with the presence of ZnT8A did not alter risk of having type 1 diabetes, and the 53 type 1 diabetes risk loci did not influence positivity for ZnT8A, despite them being disease specific.

**Conclusions/interpretation:**

ZnT8A are not primary pathogenic factors in type 1 diabetes. Nevertheless, ZnT8A testing in combination with other autoantibodies facilitates disease prediction, despite the biomarker not being under the same genetic control as the disease.

**Electronic supplementary material:**

The online version of this article (doi:10.1007/s00125-012-2540-2) contains peer-reviewed but unedited supplementary material, which is available to authorised users.

## Introduction

Autoantibodies to glutamic acid decarboxylase (GADA), insulinoma-associated antigen-2 (IA-2A) and insulin (IAA) and islet cell autoantibodies (ICA) have long been established as associated with the development of type 1 diabetes, with over 90% of newly diagnosed cases positive for at least one of these autoantibodies [1–4]. Most recently, autoantibodies to the pancreatic beta cell-specific protein, zinc transporter 8 (ZnT8A), have also been shown to be associated with type 1 diabetes [5]. Over 60% of newly diagnosed cases are positive for ZnT8A, and 4% of cases are positive for ZnT8A only [5–7], thus suggesting their utility as a predictive and diagnostic marker in type 1 diabetes [8–10]. The percentage of newly diagnosed cases positive for ZnT8A (as well as the magnitude of the ZnT8A titre) has been positively correlated with an older age at diagnosis of type 1 diabetes in children [5, 11] and has been shown to decline following diagnosis of type 1 diabetes [12, 13]. The same correlations have also been found with IA-2A and GADA [14].

The ZnT8 protein is encoded by the *SLC30A8* gene, which is convincingly associated with type 2 diabetes risk at the single nucleotide polymorphism (SNP) rs13266634C>T, an amino acid substitution R325W [15–17]. Although this amino acid variant determines the epitope specificity of ZnT8A in type 1 diabetes, either ZnT8RA or ZnT8WA [8, 11], its association with risk of the disease itself is less clear [18–20].

ZnT8RA have been reported as associated with HLA-DQB1*0302 genotypes [7] and both ZnT8RA and ZnT8WA have been correlated with HLA-DQB1*0604 haplotypes [8]. No other genetic associations have been reported with ZnT8A to date. Given the association of ZnT8A with type 1 diabetes, the 52 non-HLA regions of the genome associated with type 1 diabetes (www.t1dbase.org, accessed 13 December 2011), have a high prior probability of also being associated with ZnT8A positivity. Therefore, we measured ZnT8A (R and W epitopes) in 2,239 British individuals with type 1 diabetes and tested the known type 1 diabetes regions for association with presence of ZnT8A. A genome-wide association study (GWAS) was also performed to further test for genetic determinants of ZnT8A. Finally, we re-examined the association of rs13266634 in *SLC30A8* with ZnT8A and type 1 diabetes.

## Methods

### Samples

A total of 2,239 British type 1 diabetic individuals diagnosed before the age of 17 years were included in the study (Table 1). These constitute a randomly selected subset of the full collection of 8,000 cases that have been described elsewhere [14]. An independent dataset of 855 family members within 2 years of diagnosis of type 1 diabetes (average age at diagnosis of 12.0 years) was taken from the type 1 diabetes genetics consortium (T1DGC) affected sib-pair families and have been described previously [21]. All samples were of white European ancestry.

**Table 1:** Phenotypes of the type 1 diabetic cases by positivity for ZnT8A status

Variable	Cases overall	ZnT8A		ZnT8WA	ZnT8RA
		Positive	Negative	Positive	Positive
*n*, all samples	2,239	752	1,487	531	642
Age at diagnosis					
Mean (SD)/year	7.57 (3.91)	8.99 (3.36)	6.86 (3.98)	9.14 (3.37)	9.08 (3.31)
Median/year	7	9	7	10	9.5
Duration of diabetes					
Mean (SD)/year	6.61 (7.53)	3.45 (3.83)	8.27 (8.54)	3.38 (3.84)	3.34 (3.60)
Median/year	4	2	6	2	2
Age					
Mean (SD)/year	14.18 (8.05)	12.43 (4.75)	15.13 (9.29)	12.52 (4.70)	12.41 (4.53)
Median/year	13	12	13	13	12
Sex					
Male	1,119 (0.50)	368 (0.49)	751 (0.51)	270 (0.51)	319 (0.50)
Female	1,119 (0.50)	383 (0.51)	736 (0.49)	261 (0.49)	322 (0.50)

### Autoantibody measurement

ZnT8A were measured in serum by protein A radiobinding assays as previously described [20] using COOH-terminal (aa268-369) constructs of the ZnT8 R325 (ZnT8RA) and W325 (ZnT8WA) variants, respectively. cDNAs for the preparation of radioligands by in vitro transcription–translation were kind gifts of V. Lampasona (San Raffaele Scientific Institute, Milan, Italy). Samples were considered ZnT8A positive if antibodies to at least one of the ZnT8 variants (ZnT8RA and/or ZnT8WA) were found. Performance in the Diabetes Antibody Standardization Program 2009 workshop is shown as ‘laboratory 121’ in a published report [22]. Details of the GADA and IA-2A assays, and the ZnT8A assay in T1DGC families, have been reported previously [14, 21, 22].

### Genotyping

The *HLA-DRB1*, *HLA-DQB1*, *HLA-A*, *HLA-B* and *HLA-C* genes were typed at four digit resolution using Dynal RELI SSO assays (Invitrogen, Paisley, UK) as detailed elsewhere [23]. A subset of cases was genotyped at *HLA-DRB1* and *HLA-DQB1* using Roche Molecular Systems SSO reverse dot blot technology (Roche Molecular Systems, Pleasanton, CA, USA). The T1DGC GWAS genotyped 487,592 SNPs that passed quality control checks (2,420 within the extended MHC) using the Illumina 550K Infinium platform [24] (Illumina, San Diego, CA, USA); and 362,838 SNPs (1,687 within the extended MHC) study were genotyped using the Affymetrix GeneChip Human Mapping 500K Array set (Affymetrix, Santa Clara, CA, USA) for the Wellcome Trust Case Control Consortium (WTCCC) GWAS [25]; 75,181 were common to both platforms. Follow-up SNPs were genotyped blind to ZnT8A status using the TaqMan 5′ nuclease assay (Applied Biosystems, Warrington, UK) according to the manufacturer's protocols. All genotyping in the family samples used TaqMan. Two operators scored genotypes blind to disease status.

### Statistics

All statistical analyses were performed in R (www.r-project.org, accessed 13 December 2011) [26] or STATA (www.stata.com, accessed 13 December 2011). Association with ZnT8A (including R and W epitopes) was assessed using logistic regression models, with autoantibody positivity as the outcome variable and genotype as the predictor. Covariates, age at diagnosis and duration of diabetes were included in the regression models to account for the decrease in positivity for ZnT8A with duration of diabetes and the increase in the number of cases positive for ZnT8A with increasing age at diagnosis. For family analyses, robust variance estimates were used to account for non-independence within families [27]. Missing genotypes were imputed in the GWAS datasets using a common set of 1,500 controls genotyped on both platforms as reported previously [24]. Imputed genotypes were weighted by their posterior expectation in the regression models. To assess which autoantibody was associated with rs7522061, ZnT8A and IA-2A were used as predictors of rs7522061 genotype in regression models.

## Results

ZnT8A was measured in 2,239 British type 1 diabetic individuals. Cases positive for ZnT8A were associated with an older age at diagnosis (*p* = 3.12 × 10^−16^) and a shorter duration of diabetes (*p* = 1.10 × 10^−57^), as were both the ZnT8RA and ZnT8WA epitopes (*p* < 5 × 10^−14^; Table 1). Whilst the highest positivity for ZnT8A was observed in cases with the shortest duration of disease (≤2 years), 10% of cases who had had diabetes for ≥9 years were still positive for ZnT8A (Table 2). Therefore, both age at diagnosis and duration of diabetes were included as covariates in all tests of association with positivity for ZnT8A. This accounts for the decrease in positivity for ZnT8A with duration of disease and earlier age at diagnosis in the statistical association tests, such that all positive associations obtained can be attributed to the genetic variant tested.

**Table 2:** Positivity for ZnT8A in type 1 diabetes cases by quartiles of the duration of diabetes distribution

Duration	ZnT8A positive	ZnT8WA positive	ZnT8RA positive	ZnT8A negative
	*n* (%)	*n* (%)	*n* (%)	*n* (%)
British cases				
≤2 years	389 (58)	283 (42)	340 (51)	279 (42)
3–4 years	187 (38)	128 (26)	158 (32)	301 (62)
5–8 years	122 (22)	81 (15)	104 (19)	426 (78)
≥9 years	54 (10)	39 (7)	40 (8)	479 (90)
T1DGC families				
<2 years	523 (60)	412 (54)	462 (61)	345 (40)

*n* number of samples

A subset of 1,307 cases with ZnT8A measurements had previously been tested for GADA and IA-2A [14, 21]. Of these, just 277 (21%) were negative for all three autoantibodies and 28 were only positive for ZnT8A. There was a strong association of positivity for ZnT8A with both IA-2A and GADA positivity, after accounting for age at diagnosis and duration of disease (OR 4.45 [95% CI 3.32, 5.99] *p* = 8.46 × 10^−26^ and OR 1.63 [95% CI 1.24, 2.14] *p* = 5.04 × 10^−4^, respectively); 81% of ZnT8A positives were also positive for IA-2A, whereas 63% were positive for GADA.

### ZnT8A association in type 1 diabetes regions

**Fig. 1:**
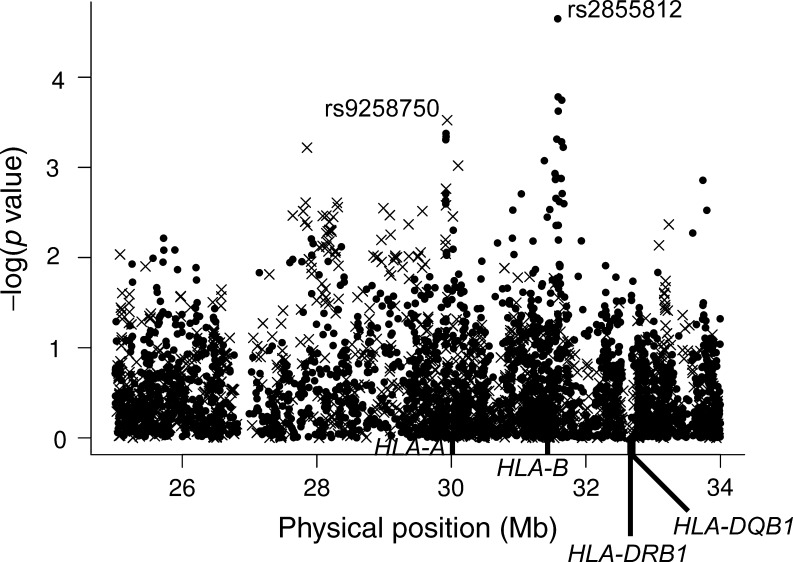
Association of ZnT8A with SNPs in the MHC region of chromosome 6. SNPs are analysed in up to 1,021 type 1 diabetes cases at the WTCCC SNPs (crosses) and up to 1,123 type 1 diabetes cases at the T1DGC SNPs (dots). −log(*p* value for association with ZnT8A positivity) is plotted against physical position in megabases (Mb). The physical locations of the HLA classical loci are also given

Outside of the HLA, no evidence of association with positivity for ZnT8A was obtained in the type 1 diabetes associated regions with the British cases (*p* > 0.03). None of the classical HLA loci, *HLA-DRB1*, *HLA-DQB1*, *HLA-A*, *HLA-B* and *HLA-C*, were associated with ZnT8A (*p* > 0.05; Fig. 1). Evidence of association in the HLA region mapped to the class I region (Fig. 1). The most strongly associated SNP was rs2855812 (*p* = 2.25 × 10^−5^) in *MICB* in the T1DGC cases, and rs9258750 (*p* = 3.00 × 10^−4^) near *HLA-A* in the WTCCC cases. The genotyping of these SNPs was extended to over 2,100 cases in which ZnT8A was measured (electronic supplementary material [ESM] Table 1). The rs9258750 SNP showed more evidence of association with positivity for ZnT8A (*p* = 2.06 × 10^−9^; OR for the major A allele 1.82 [95% CI 1.49, 2.23]) than rs2855812 (*p* = 5.80 × 10^−5^; OR for the minor T allele 1.33 [95% CI 1.16, 1.53]). The two SNPs were not in linkage disequilibrium (LD) (*r* ^2^ = 0.02, D′ = 0.33) and hence appear to mark independent effects (*p* = 7.2 × 10^−4^ for the addition of rs2855812 to rs9258750, and *p* = 5.83 × 10^−8^ for the addition of rs9258750 to rs2855812). rs9258750 was in LD with HLA-A*24 (*r* ^2^ = 0.54, D′ = 0.98). HLA-A*24 was associated with ZnT8A positivity (*p* = 2.03 × 10^−4^), but this was due to rs9258750: *p* = 0.95 for the addition of HLA-A*24 to rs9258750, compared with *p* = 0.0015 for the addition of rs9258750 to HLA-A*24 in regression models. The association of rs9258750 with positivity for ZnT8A was confirmed in an independent family dataset of white European ancestry (*p* = 0.0014; OR for the major A allele 1.57 [95% CI 1.19, 2.06] in the 833 affected family members successfully genotyped).

### Genome-wide association

**Fig. 2:**
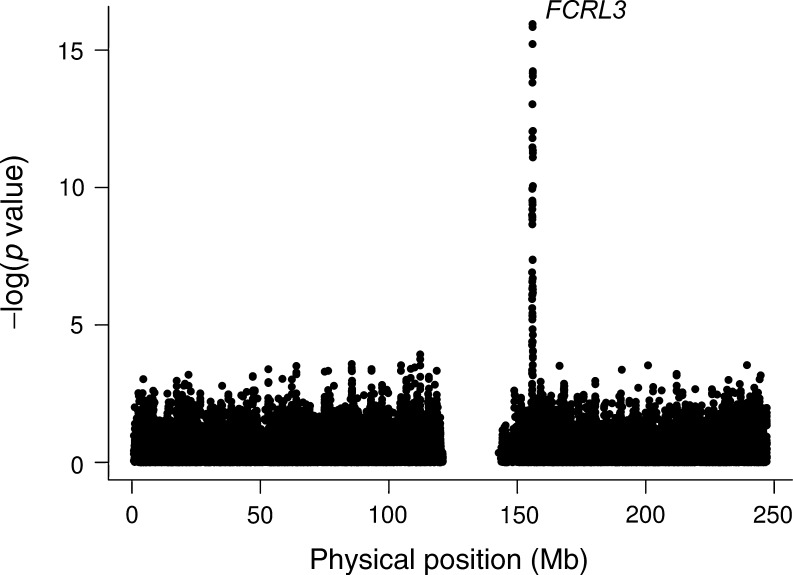
Association of positivity for ZnT8A with SNPs on chromosome 1 in up to 2,142 type 1 diabetes cases

Having found no evidence of association with positivity for ZnT8A in the type 1 diabetes associated regions, the search was extended genome wide. Up to a maximum of 2,142 cases were analysed at 775,249 SNPs across the genome. Only the *FCRL3* gene region on chromosome 1 attained significance at a GWAS level (*p* < 5 × 10^−8^) with all SNPs outside of this region having *p* values above 1 × 10^−6^ (Fig. 2 and ESM Fig. 1). The most associated SNP, rs7522061, (*p* = 1.13 × 10^−16^) is located in exon 4 of the *FCRL3* gene (Ensembl version 63) and is in LD with rs7528684 (*r* ^2^ = 0.89 in British controls), which has previously been shown to be associated with IA-2A in type 1 diabetes cases [21, 28]. The region is also associated with Graves' disease and thyroid peroxidase autoantibody positivity at rs11264798 [21, 25].

Neither rs7528684 (the IA-2A associated variant) nor rs11264798 (the Graves' disease associated variant) were genotyped as part of the GWAS, but had been genotyped previously using TaqMan [21]. Both SNPs were associated with positivity for ZnT8A (rs7528684, *p* = 1.79 × 10^−16^; OR for the major A allele 1.81 [95% CI 1.56, 2.09] and rs11264798 *p* = 4.86 × 10^−14^; OR for the C allele 1.72 [95% CI 1.48, 1.98]). Only the WTCCC samples were genotyped at rs7522061 (with imputation used for the remaining samples in the GWAS), and so this was extended to 2,131 cases by TaqMan genotyping (Table 3). Whilst rs7522061 accounted for the association at rs11264798, both rs7522061 and rs7528684 appear to be marking the same association, as their effects could not be distinguished in the 2,088 cases genotyped at both loci: rs7528684 did not improve a model with rs7522061 (*p* = 0.15) and conversely, rs7522061 did not improve a model with rs7528684 (*p* = 0.10). Given that IA-2A and ZnT8A are correlated, we tested whether the association with positivity for ZnT8A at *FCRL3* was due to the known association with IA-2A. Using 1,241 cases in a model that included both ZnT8A and IA-2A, positivity for ZnT8A predicted the rs7522061 genotype (*p* = 5.51 × 10^−11^) and IA-2A positivity did not (*p* = 0.10).

**Table 3:** Association of positivity for ZnT8A with rs7522061 T > C in *FCRL3*

rs7522061	T allele			*p* value
	*n* (frequency)		OR [95% CI]	
	Positive	Negative		
British cases				
ZnT8A	876 (0.61)	1,395 (0.49)	1.82 [1.57, 2.10]	1.13 × 10^−16^
ZnT8WA	630 (0.62)	1,641 (0.51)	1.70 [1.45, 1.98]	8.13 × 10^−12^
ZnT8RA	735 (0.61)	1,536 (0.50)	1.65 [1.42, 1.91]	1.74 × 10^−11^
Family samples				
ZnT8A	601 (0.59)	322 (0.49)	1.50 [1.22, 1.84]	1.20 × 10^−4^

The minor allele is negatively associated with the presence of ZnT8A in both the British cases and the family samples, so ORs and allele frequencies are given for the major T allele. The association of rs7522061 with ZnT8A is consistent across both ZnT8WA and ZnT8RA epitopes

The association at *FCRL3* was confirmed in up to 855 affected family members of white European ancestry. Confirmation of association with ZnT8A was obtained at rs7522061, rs7528684 and rs11264798 (*p* = 1.20 × 10^−4^, 3.14 × 10^−4^ and 9.20 × 10^−4^, respectively) with the same direction of effect as in the British cases (OR for the T allele at rs7522061, 1.50 [95% CI 1.22, 1.84]; Table 3).

### Association of ZnT8A with *SLC30A8*

In the GWAS, whilst evidence of association with positivity for ZnT8A was obtained at rs13266634/R325W in *SLC30A8* (*p* = 5.29 × 10^−5^), it was not at a level required for GWAS significance. As genotypes were only available for those samples genotyped using the Illumina platform, rs13266634 was genotyped in all samples using TaqMan (as detailed in the Methods). The SNP was associated with positivity for ZnT8A (*p* = 3.66 × 10^−11^). However, a multiplicative allelic effects model did not fit the data. The minor T/T homozygous genotype was associated with ZnT8A positivity and the heterozygous C/T genotype with absence of ZnT8A (Table 4). Analysis of the two epitopes, ZnT8WA and ZnT8RA, revealed that the T allele at rs13266634 was associated with ZnT8WA positivity (*p* = 9.26 × 10^−27^) and ZnT8RA negativity (*p* = 7.20 × 10^−17^; Table 4). This inverse association of the two ZnT8A epitopes with rs13266634 accounted fully for the deviation from a multiplicative allelic effects model with positivity for ZnT8A overall. Importantly, this SNP is not associated with type 1 diabetes overall (*p* = 0.15 in 7,680 British type 1 diabetes cases and 7,200 British controls; ESM Table 2).

**Table 4:** Association of rs13266634 in *SLC30A8* with positivity for ZnT8A in 2,191 type 1 diabetes cases

rs13266634	ZnT8A			ZnT8WA			ZnT8RA		
	*n* (frequency)		OR [95% CI]	*n* (frequency)		OR [95% CI]	*n* (frequency)		OR [95% CI]
	Positive	Negative		Positive	Negative		Positive	Negative	
T	500 (0.34)	928 (0.32)	1.07 [0.93, 1.24]	478 (0.46)	950 (0.28)	2.38 [2.02, 2.80]	310 (0.25)	1,118 (0.36)	0.51 [0.43, 0.60]
C/C	348 (0.47)	651 (0.45)	1.00 (reference)	152 (0.29)	847 (0.51)	1.00 (reference)	348 (0.56)	651 (0.42)	1.00 (reference)
C/T	272 (0.37)	684 (0.47)	0.64 [0.52, 0.78]	254 (0.49)	702 (0.42)	2.01 [1.59, 2.55]	246 (0.39)	710 (0.45)	0.54 [0.44, 0.67]
T/T	114 (0.16)	122 (0.08)	1.90 [1.38, 2.62]	112 (0.22)	124 (0.07)	6.31 [4.48, 8.88]	32 (0.05)	204 (0.13)	0.23 [0.15, 0.35]
*p* value			3.66 × 10^−11 a^			9.26 × 10^−27 b^			7.20 × 10^−17 b^

*n* number of alleles or genotypes

^a^Note that the multiplicative model did not fit the data, *p* = 6.44 × 10^−12^, hence the *p* value reported is for a model that does not assume a specific mode of inheritance

^b^Note the multiplicative model was an appropriate approximation for the association with ZnT8WA (*p* = 0.056) and ZnT8RA (*p* = 0.38)

## Discussion

We have obtained convincing evidence of association between positivity for ZnT8A and the nonsynonymous SNP, rs7522061, in *FCRL3*, and in our datasets this association was indistinguishable from the association with the non-coding SNP, rs7528684. The T allele of rs7522061, which is associated with positivity for ZnT8A, alters the amino acid sequence (Asp to Asn), which could alter protein function and/or RNA stability/splicing. Only in relation to multiple sclerosis have there been reports of this SNP being associated; the same study also reported disease association with rs7528684 [29]. The minor G allele at rs7528684 is associated with risk of rheumatoid arthritis and systemic lupus erythematosus [30], protection from autoimmune Addison disease [31] and multiple sclerosis [29], and has been found to alter the binding affinity of nuclear factor-κΒ [30], making it a good functional candidate SNP. Interestingly, *FCRL3* is not associated with type 1 diabetes risk [21], even though it is strongly associated with IA-2A positivity [21, 28]. We have shown here that this association is probably due to a primary association with positivity for ZnT8A and not with IA-2A positivity directly. The association with positivity for ZnT8A was marked equally well by rs7522061 or rs7528684, and consequently, further work is required to unravel the precise nature of the association with the presence of ZnT8A in the *FCRL3* region and the mechanisms involved.

None of the regions known to be associated with type 1 diabetes, outside of the HLA, were associated with positivity for ZnT8A. Interestingly, evidence of association with positivity for ZnT8A was confined to the HLA class I region, but the most associated variant, rs9258750, was not associated with type 1 diabetes (*p* = 0.13 in 8,232 British cases and 9,757 British controls; data not shown). The known associations of IA-2A with HLA-A*24 [14, 32] and of GADA with rs9266722 in HLA class I [14] were independent of the ZnT8A associations described in the present study. In contrast to previous reports in smaller sample sets, no association with HLA-DQB1*0302, *0604, *04 alleles and ZnT8A was obtained (*p* > 0.15), neither with positivity for ZnT8RA (*p* > 0.15) nor ZnT8WA (*p* > 0.16).

Our study is the largest of its kind to date, using 2,239 case samples, 70% of whom had had ZnT8A measured, having been diagnosed with type 1 diabetes more than 2 years earlier. Therefore, we have been able to confirm recent reports that showed that positivity for ZnT8A declines after diagnosis of disease [12, 13]. Approximately 40% of cases tested for ZnT8A who have had diabetes for 4 years are expected to test positive compared with 62% at diagnosis [12]. In our samples it was comparable (Table 2). A consequence of this decline in positivity for ZnT8A with duration of diabetes is that the rate of ZnT8A negative samples will be elevated amongst cases with longer disease duration. Consequently, almost half the cases classed as ZnT8A negative who had had diabetes for 9 or more years would probably have tested positive had they been tested at diagnosis. This causes the association signal at some loci to be diluted in these samples, but does not affect the validity of the positive associations detected. The *HLA* and *FCRL3* associations with ZnT8A in cases who have had diabetes for ≤2 years are of comparable strength to those who have had diabetes for ≥9 years (ESM Tables 3 and 4). All positive associations were confirmed in the cases from the families affected by type 1 diabetes, all of whom were tested within 2 years of diagnosis. The lack of association outside of the HLA and *FCRL3* may be attributable to lack of power, which was exacerbated by the increased frequency of ZnT8A negativity amongst samples with a longer duration of disease. The inverse correlation of positivity for ZnT8A with duration of diabetes was not known when we designed our study, and hence, the statistical power of future studies into the genetic architecture of presence of ZnT8A would benefit from using cases as close to diagnosis as possible, as we have done for the replication in family samples.

For autoantibodies to be causal for type 1 diabetes, the variants associated with risk of type 1 diabetes would also be expected to be associated with autoantibody positivity. Our results, therefore, indicate that autoantibodies to ZnT8 are a downstream event to the primary genetic pathogenesis of type 1 diabetes. Given their specificity for type 1 diabetes [6], however, this does not detract from their well established value, in combination with other autoantibodies, in assessing risk of the disease [6, 10, 20], but does illustrate how useful biomarkers need not necessarily have associations with genetic markers that confer susceptibility to disease.

## Electronic supplementary material

Below is the link to the electronic supplementary material.

ESM Table 1 (PDF 16.4 kb)

ESM Table 2 (PDF 12.6 kb)

ESM Table 3 (PDF 22.0 kb)

ESM Table 4 (PDF 22.4 kb)

ESM Figure 1 (PDF 2,254 kb)

## Abbreviations

GWAS: Genome-wide association study
IA-2A: Autoantibodies to insulinoma-associated antigen-2
IAA: Autoantibodies to insulin
ICA: Islet cell autoantibodies
LD: Linkage disequilibrium
SNP: Single nucleotide polymorphism
T1DGC: Type 1 diabetes genetics consortium
WTCCC: Wellcome Trust Case Control Consortium
ZnT8A: Autoantibodies to zinc transporter 8
