# Supplementary material for: Genetic association of zinc transporter 8 (ZnT8) autoantibodies in type 1 diabetes cases
Source: Diabetologia. 2012 Apr 12;55(7):1978–84. doi: 10.1007/s00125-012-2540-2 (PMC3369141; doi:10.1007/s00125-012-2540-2)
Supplement: Supplementary file 1 — (PDF 16.4 kb) [file 125_2012_2540_MOESM1_ESM.pdf]

ESM Table 1 Association of the HLA class I SNPs, rs9258750A>G and rs2855812G>T with positivity for ZnT8A. The minor G allele at rs9258750 is negatively associated with ZnT8A.

rs9258750

**Allele or genotype**	**n(frequency)**		**OR [95% CI]**		***p*-value**
	**ZnT8A positives**	**ZnT8A negatives**			
**A**	1,258 (0.88)	2,269 (0.80)	1.82 [1.49-2.23]		2.06×10^{-9}
**A/A**	547 (0.76)	903 (0.64)	6.31 [2.42-16.41]	1.00 [reference][a]	
**A/G**	164 (0.23)	463 (0.33)	3.75 [1.42-9.86]	0.59 [0.47-0.74][a]	
**G/G**	5 (0.01)	46 (0.03)	1.00 [reference]	0.16 [0.06-0.41][a]	

rs2855812

**Allele or genotype**	**n(frequency)**		**OR [95% CI]**	***p*-value**
	**ZnT8A positives**	**ZnT8A negatives**		
**T**	639 (0.45)	1138 (0.41)	1.33 [1.16-1.53]	5.80×10^{-5}
**G/G**	221 (0.31)	508 (0.36)	1.00 [reference]	
**G/T**	341 (0.48)	640 (0.46)	1.44 [1.15-1.80]	
**T/T**	149 (0.21)	249 (0.18)	1.73 [1.30-2.30]	

[a] ORs using the common A/A genotype as reference are also given as they have tighter 95% confidence intervals than those using the rare G/G genotype as reference.
Both SNPs were in Hardy-Weinberg equilibrium in controls ($p > 0.2$).
