# Supplementary material for: Genetic association of zinc transporter 8 (ZnT8) autoantibodies in type 1 diabetes cases
Source: Diabetologia. 2012 Apr 12;55(7):1978–84. doi: 10.1007/s00125-012-2540-2 (PMC3369141; doi:10.1007/s00125-012-2540-2)
Supplement: Supplementary file 2 — (PDF 12.6 kb) [file 125_2012_2540_MOESM2_ESM.pdf]

ESM Table 2 Association analysis of the *SLC30A8* SNP, rs13266634 in 7,680 British type 1 diabetes cases and 7,200 British controls.

Allele or Genotype	n (frequency)		OR [95% CI]	*p*-value
	Cases	Controls		
**T**	4,879 (0.32)	4,463 (0.31)	1.04 [0.99-1.09]	0.15
**C/C**	3,605 (0.47)	3,445 (0.48)	1.00 [reference]	
**C/T**	3,271 (0.43)	3,047 (0.42)	1.03 [0.96-1.10]	
**T/T**	804 (0.10)	708 (0.10)	1.09 [0.97-1.22]	
