# Supplementary material for: Genetic association of zinc transporter 8 (ZnT8) autoantibodies in type 1 diabetes cases
Source: Diabetologia. 2012 Apr 12;55(7):1978–84. doi: 10.1007/s00125-012-2540-2 (PMC3369141; doi:10.1007/s00125-012-2540-2)
Supplement: Supplementary file 3 — (PDF 22.0 kb) [file 125_2012_2540_MOESM3_ESM.pdf]

ESM Table 3 Frequency of the major A allele at the HLA class I SNP, rs9258750, by positivity for ZnT8A and quartiles of the duration of diabetes distribution. The association with positivity for ZnT8A is in the same direction for all categories of the duration of diabetes distribution and surprisingly, there is little difference in allele frequency as duration of diabetes increases, either within cases positive for ZnT8A or within those negative for ZnT8A.

Duration of diabetes / years	n (frequency)		
	ZnT8A positives	ZnT8A negatives	All cases (n=2,128)
≤ 2	650 (0.87)	436 (0.81)	1086 (0.85)
3-4	320 (0.89)	460 (0.79)	780 (0.83)
5-8	207 (0.88)	633 (0.80)	840 (0.82)
≥ 9	81 (0.86)	740 (0.81)	821 (0.82)

n ~ number
