# Supplementary material for: Genetic association of zinc transporter 8 (ZnT8) autoantibodies in type 1 diabetes cases
Source: Diabetologia. 2012 Apr 12;55(7):1978–84. doi: 10.1007/s00125-012-2540-2 (PMC3369141; doi:10.1007/s00125-012-2540-2)
Supplement: Supplementary file 4 — (PDF 22.4 kb) [file 125_2012_2540_MOESM4_ESM.pdf]

ESM Table 4 Frequency of the major T allele at the *FCRL3* SNP, rs7522061 by positivity for ZnT8A and quartiles of the duration of diabetes distribution. As expected the frequency of the T allele is elevated in cases negative for ZnT8A beyond 2 years of diagnosis compared to those tested within two years of diagnosis. This is consistent with some cases being categorised as negative for ZnT8A at time of testing, who may have tested positive for ZnT8A had they been tested at diagnosis. Nevertheless, the association with positivity for ZnT8A is in the same direction for each of the duration of diabetes groups.

Duration of diabetes / years	n (frequency)		
	ZnT8A positives	ZnT8A negatives	All cases (n=2,131)
≤ 2	434 (0.59)	236 (0.44)	670 (0.52)
3-4	216 (0.61)	291 (0.51)	507 (0.55)
5-8	149 (0.64)	402 (0.50)	551 (0.53)
≥ 9	77 (0.73)	466 (0.51)	543 (0.53)

n ~ number
