# Supplementary material for: Genetic association of zinc transporter 8 (ZnT8) autoantibodies in type 1 diabetes cases
Source: Diabetologia. 2012 Apr 12;55(7):1978–84. doi: 10.1007/s00125-012-2540-2 (PMC3369141; doi:10.1007/s00125-012-2540-2)
Supplement: Supplementary file 5 — (PDF 2,254 kb) [file 125_2012_2540_MOESM5_ESM.pdf]

ESM Fig. 1 Results of a genome-wide association study of positivity for ZnT8A in up to 2,142 type 1 diabetes cases. –log *p*-value is plotted against physical position on a given chromosome in mega bases (Mb). Note chromosome 1 is given in Fig. 2 and chromosome 6 does not include the HLA region, which is given in Fig. 1.

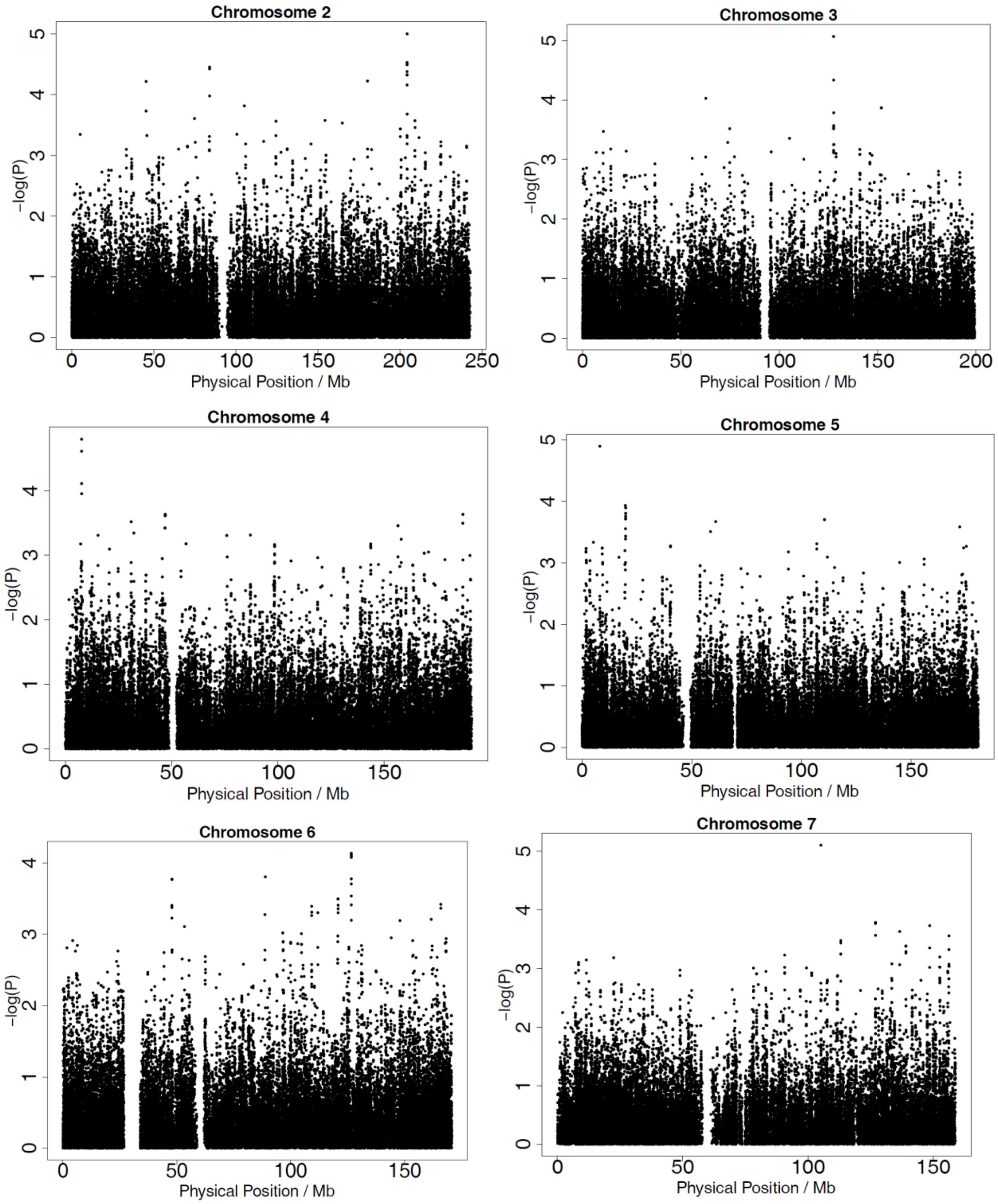

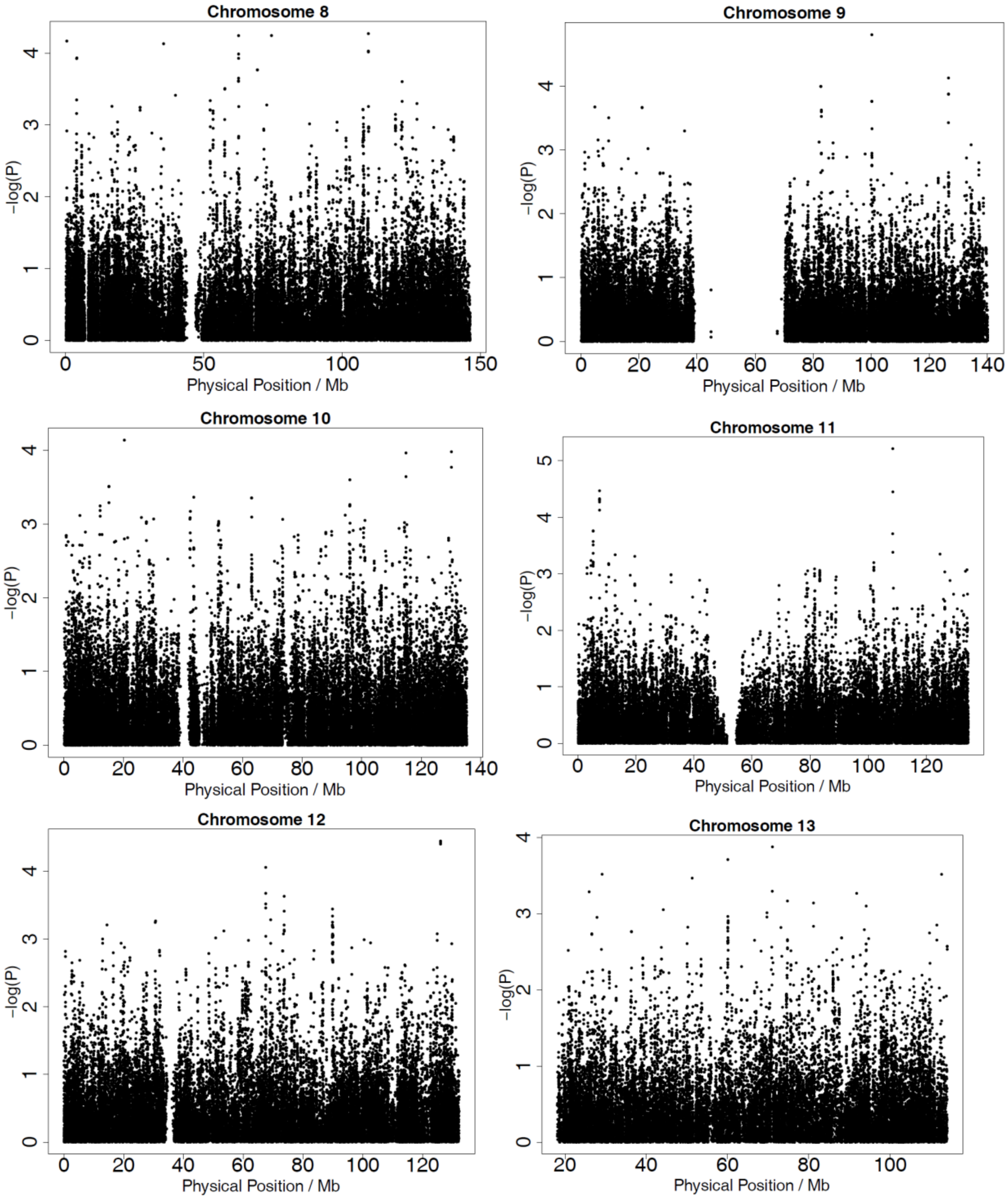
Chromosome 8
−log(P)
Physical Position / Mb
Chromosome 9
−log(P)
Physical Position / Mb
Chromosome 10
−log(P)
Physical Position / Mb
Chromosome 11
−log(P)
Physical Position / Mb
Chromosome 12
−log(P)
Physical Position / Mb
Chromosome 13
−log(P)
Physical Position / Mb

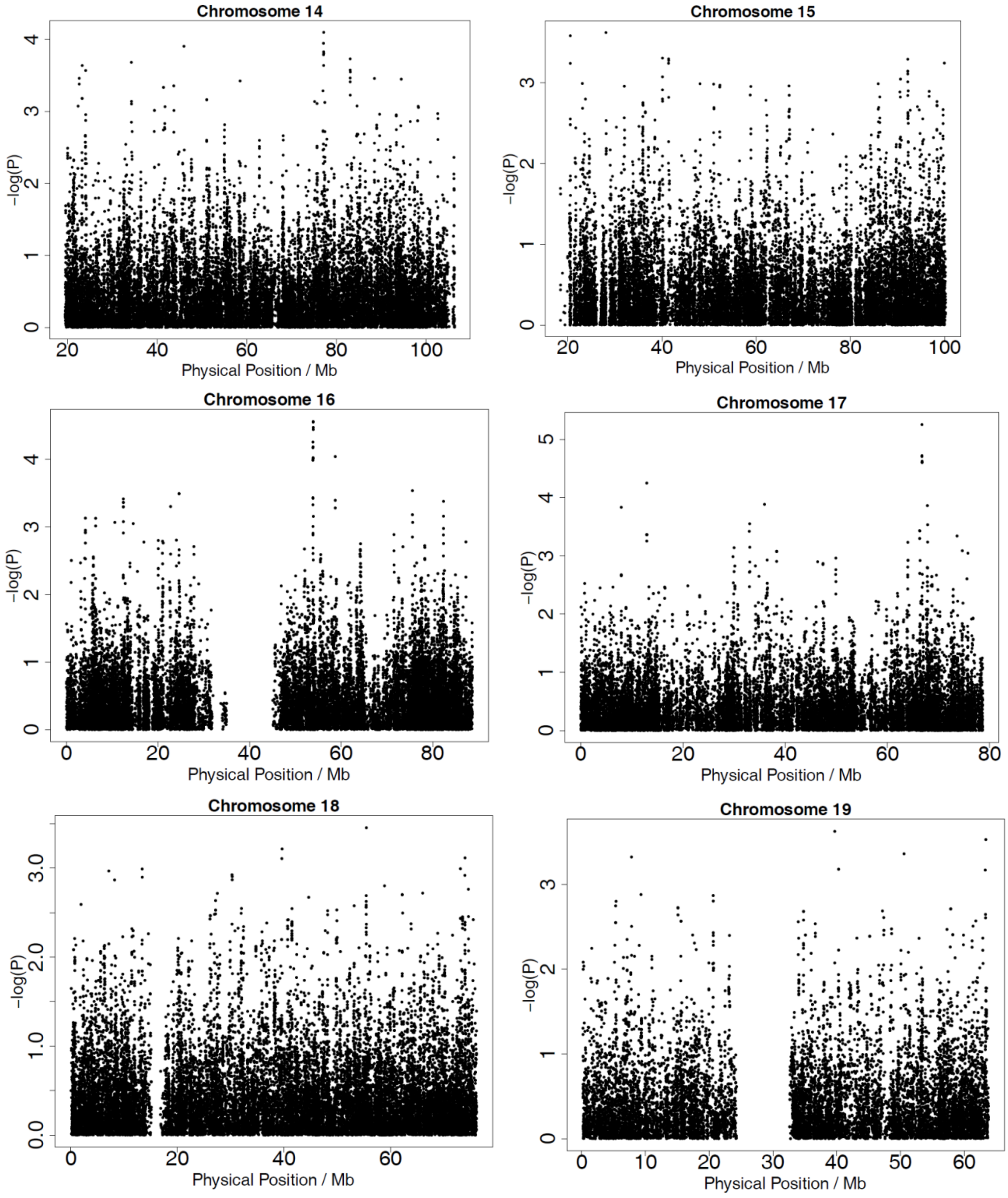
Chromosome 14
Chromosome 15
Chromosome 16
Chromosome 17
Chromosome 18
Chromosome 19
−log(P)
Physical Position / Mb

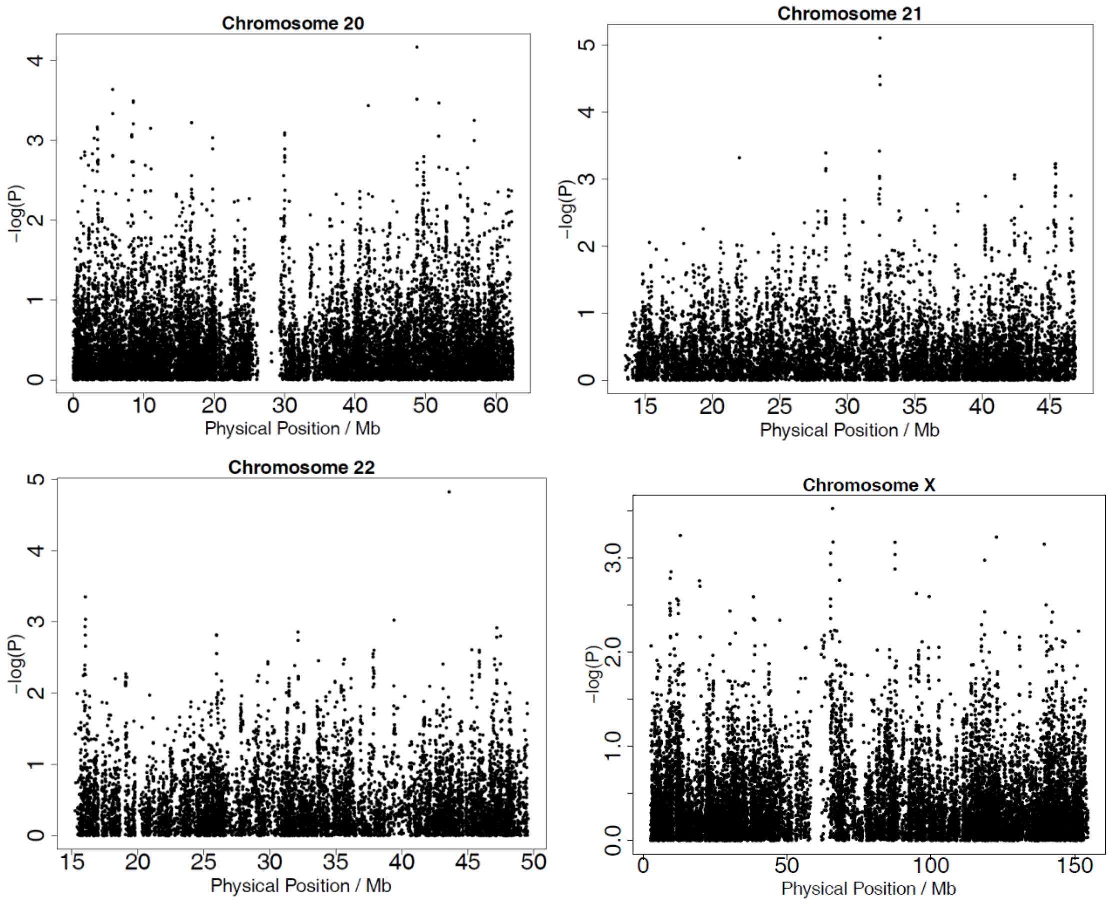
Chromosome 20
Chromosome 21
Chromosome 22
Chromosome X
-log(P)
Physical Position / Mb
